# Methyl coenzyme M reductase (*mcrA*) gene abundance correlates with activity measurements of methanogenic H_2_/CO_2_-enriched anaerobic biomass

**DOI:** 10.1111/1751-7915.12094

**Published:** 2013-10-31

**Authors:** Rachel Morris, Anne Schauer-Gimenez, Ujwal Bhattad, Colleen Kearney, Craig A Struble, Daniel Zitomer, James S Maki

**Affiliations:** 1 Department of Biological Sciences, Marquette UniversityMilwaukee, WI, 53201-1881, USA; 2 Department of Civil, Construction and Environmental Engineering, Water Quality Center, Marquette UniversityMilwaukee, WI, 53201-1881, USA; 3 Department of Mathematics, Statistics and Computer Science, Marquette UniversityMilwaukee, WI, 53201-1881, USA

## Abstract

Biologically produced methane (CH_4_) from anaerobic digesters is a renewable alternative to fossil fuels, but digester failure can be a serious problem. Monitoring the microbial community within the digester could provide valuable information about process stability because this technology is dependent upon the metabolic processes of microorganisms. A healthy methanogenic community is critical for digester function and CH_4_ production. Methanogens can be surveyed and monitored using genes and transcripts of *mcrA*, which encodes the α subunit of methyl coenzyme M reductase – the enzyme that catalyses the final step in methanogenesis. Using clone libraries and quantitative polymerase chain reaction, we compared the diversity and abundance of *mcrA* genes and transcripts in four different methanogenic hydrogen/CO_2_ enrichment cultures to function, as measured by specific methanogenic activity (SMA) assays using H_2_/CO_2_. The *mcrA* gene copy number significantly correlated with CH_4_ production rates using H_2_/CO_2_, while correlations between *mcrA* transcript number and SMA were not significant. The DNA and cDNA clone libraries from all enrichments were distinctive but community diversity also did not correlate with SMA. Although hydrogenotrophic methanogens dominated these enrichments, the results indicate that this methodology should be applicable to monitoring other methanogenic communities in anaerobic digesters. Ultimately, this could lead to the engineering of digester microbial communities to produce more CH_4_ for use as renewable fuel.

## Introduction

Anaerobic wastewater treatment is an environmentally and economically beneficial process in which the biological degradation of organic wastes results in the production of CH_4_ as a carbon-neutral energy source (Zitomer *et al*., 2008). Unfortunately, the widespread application of this ‘green’ technology has been hampered by concerns about process stability. Although treatment failure can be a serious problem, monitoring of anaerobic biomass can be used to measure the efficacy of bioaugmentation or system control used to prevent digester failure or encourage faster recovery of stressed digesters (Castellano *et al*., 2007; Schauer-Gimenez *et al*., 2010).

Digester failure occurs when the microbial community is stressed by organic overload or toxicants or other abrupt environmental changes (Castellano *et al*., 2007). Therefore, the results of assays that rapidly monitor the microbial community in anaerobic biomass could provide useful information to operators seeking to manage digester function. In practice, however, the microorganisms in the anaerobic microbial community are rarely monitored directly. In fact, the microbial community in anaerobic digesters has been a black box throughout most of the history of this technology (Rivière *et al*., 2009).

Specific methanogenic activity (SMA) assays, methane production rates, biogas composition, chemical oxygen demand (COD) removal, pH, granule morphology, acetate utilization rates, methanethiol concentration and quantification of volatile fatty acids have all been suggested or used to evaluate digester function (Coates *et al*., 1996; Zitomer *et al*., 2000; Castellano *et al*., 2007; Conklin *et al*., 2008; Molina *et al*., 2009). While these parameters are closely related to the metabolic functions of the microbial community, they do not directly quantify microorganisms. Successful removal of organic waste from the influent wastewater and methane production depend upon the collaborative efforts of the members of an interdependent microbial community, so knowledge of the structure and function of the community in anaerobic wastewater digesters can be very useful when attempting to stabilize or increase the efficiency of waste removal and biogas production.

For example, SMA assays have been used to evaluate changes in biomass activity by quantifying the production of methane per the amount of volatile suspended solids (VSS) per unit time (Coates *et al*., 1996; 2005). The methanogens are the source of the methane, and they can be directly targeted using molecular microbiological methods. Methanogen genomes encode a unique operon for the enzyme methyl coenzyme M reductase (MCR). Previous studies have established that the presence and transcription of the gene for the alpha subunit of MCR (*mcrA*) can be used to detect the presence, abundance and/or activity of methanogens in natural and engineered environments (Springer *et al*., 1995; Luton *et al*., 2002; Juottonen *et al*., 2008; Gagnon *et al*., 2011; Kampmann *et al*., 2012; Munk *et al*., 2012). Other studies have demonstrated that the methane flux from peat and biogas production from anaerobic biomass correlated with the abundance of *mcrA* (Freitag and Prosser, 2009; Freitag *et al*., 2010; Traversi *et al*., 2012). Based in part on these reports, it was hypothesized that quantification of *mcrA* genes and/or transcripts by quantitative polymerase chain reaction (qPCR) would correlate with SMA results and could thus be used in their stead. The substitution could provide a substantial benefit because qPCR assays can be completed within 24 h from biomass collection – whereas SMA assays can take up to 7–10 days to complete, giving digester operators information about the activity of the biomass much more rapidly than SMA assays.

Herein, we report an evaluation of the use of qPCR of *mcrA* genes and transcripts in comparison with traditional SMA assays on the biomass from four different H_2_/CO_2_ enriched bioreactors.

## Results

### SMA assays

**Fig. 1:**
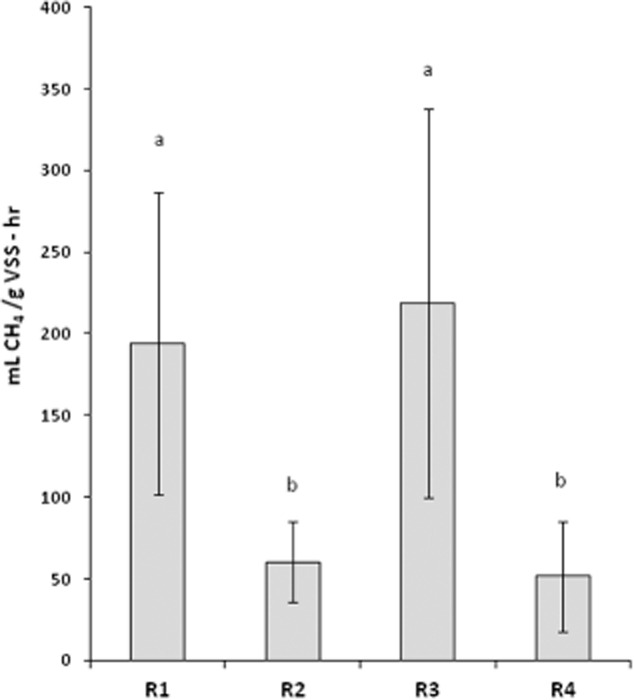
Specific methanogenic activity (SMA) against H_2_/CO_2_ (ml CH_4_ g^−1^ volatile suspended solids [VSS] -h^−1^) for each anaerobic enrichment culture (*n* = 13). SMA values from R1 and R3 were different (*P* < 0.05) from those from R2 and R4. Measurements not different from each other are indicated with the letters a or b.

SMA values for assays using H_2_/CO_2_ (13 trials with three technical replicates each) ranged from 51.8 to 218.6 ml CH_4_ g^−1^ of VSS h^−1^ (Fig. 1). Mean SMA values for cultures R1 and R3 were significantly higher than those of R2 and R4 (*P* < 0.05 Kruskal–Wallis test, nonparametric multiple comparison test, Zar, 2010). SMA values from assays using acetate or propionate (one trial each with three technical replicates each) were below detection for all cultures. Volatile fatty acids (acetic acid, propionic acid, iso-butyric acid, butyric acid, iso-valeric acid and valeric acid) were less than 50 mg ml^−1^ in each culture (one trial). Culture pH averages from 30 days of monitoring were as follows (pH ± standard deviation): R1 (7.4 ± 0.18), R2 (7.27 ± 0.12), R3 (7.36 ± 0.15) and R4 (7.32 ± 0.14).

### Quantitative PCR and reverse transcriptase PCR (RT-PCR)

qPCR was performed on DNA and RT-PCR products in a single run, and a disassociation (melt) curve was performed to check primer specificity. Critical parameters for the run were as follows: PCR efficiency, 110.5%, slope of standard curve, −3.093, y-intercept, 5.134, correlation coefficient, 0.949. The C_t_ for the no template control was 24.03 and > 26.5 for all the no-reverse transcriptase controls. The C_t_ value for the no template control can be explained by the observation of primer dimer formation; however, when template was present, no primer dimers were observed in the melt curve. All reported results were based on C_t_ values less than no-reverse transcriptase controls and were within the linear range of the standard curve.

**Fig. 2:**
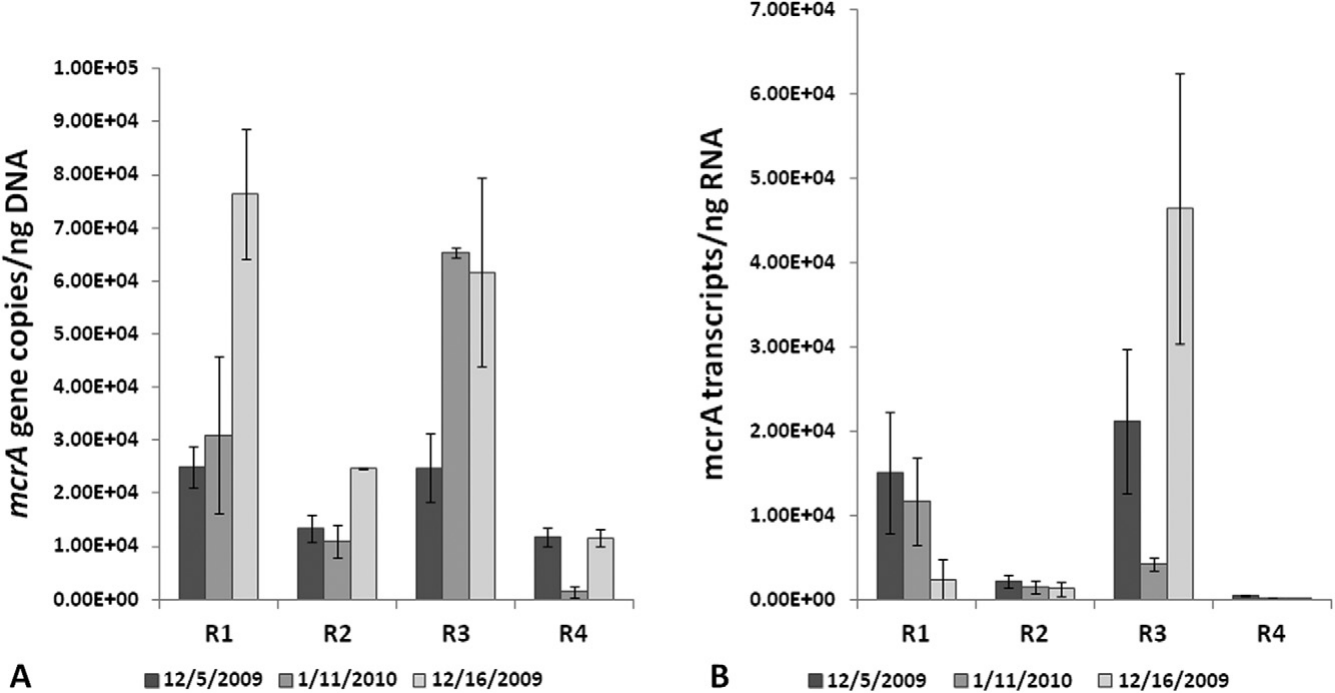
qPCR data.A. Quantification of *mcrA* gene copies ng^−1^ DNA from multiple nucleic acid extractions of bioreactor biomass.B. Quantification of *mcrA* transcripts ng^−1^ RNA from the same samples of bioreactor biomass. Bars in both panels show standard error of the mean.

When the data from each date were compared, biomass from cultures R1 and R3 had greater *mcrA* gene copy ng^−1^ DNA and transcript numbers ng^−1^ RNA than did biomass from cultures R2 and R4 (Figs 1 and 2). Variations in *mcrA* copy number and transcript number were observed among the three samples of biomass taken from each of the enrichment cultures on different dates (Fig. 2A and B). However, in spite of the variation between sampling dates, the trend of greater copy and transcript numbers in R1 and R3 remained the same. Transcript to gene copy ratios were calculated from qPCR and qRT-PCR results for each culture with the *mcrA* values after they were normalized to total nucleic acids as in Freitag and colleagues (2010).

### Comparison of qPCR and SMA

**Fig. 3:**
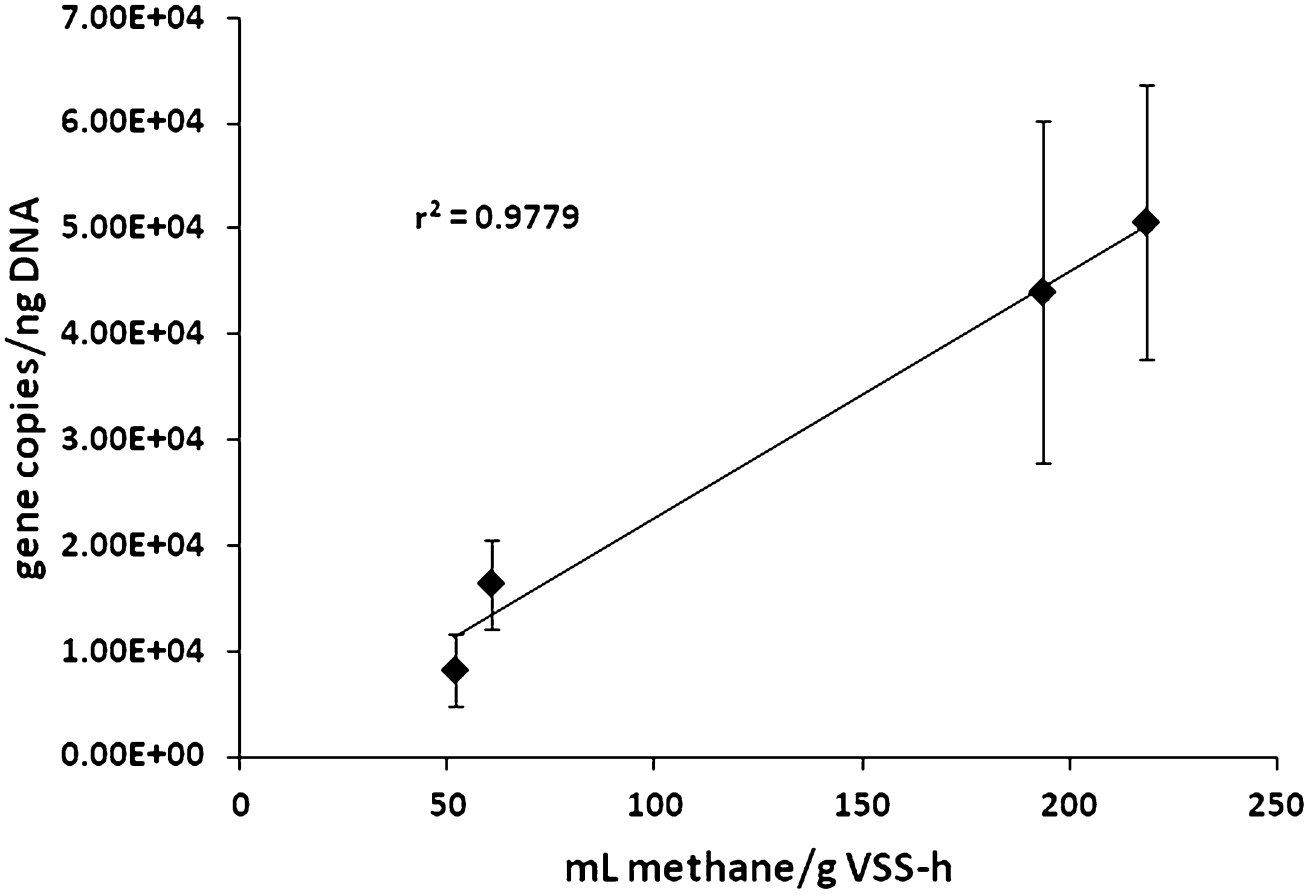
Relationship between *mcrA* gene copy ng^−1^ DNA abundance in the four bioreactors and SMA. qPCR results versus SMA were significant (r^2^ = 0.9779, *P* = 0.0074).

qPCR results from DNA extractions (*mcrA* gene copy number ng^−1^ DNA) showed significant correlation with SMA results against H_2_:CO_2_, r^2^ = 0.9779, *P* < 0.01 (Fig. 3). Transcript number correlation to SMA values was not significant (*P* = 0.099). No significant correlation was detected between *mcrA* transcript to gene copy (both values normalized to total nucleic acids) ratios and SMA values (r^2^ = 0.33, *P* > 0.05).

### Methanogen community analysis

**Fig. 4:**
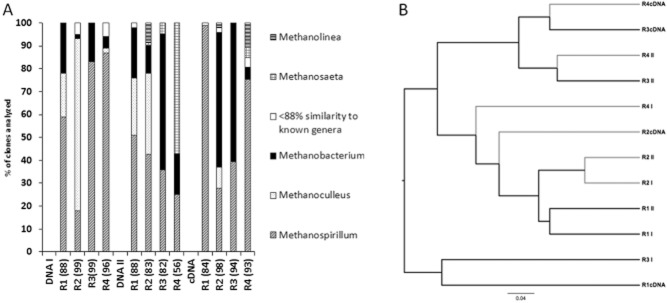
A. Methanogen genus assignments for *mcrA* clones. Relative abundance of *mcrA* clones in each library to specific methanogen genera based on 88% sequence similarity according to Steinberg and Regan (2008). B. Dendrogram using the Sorenson index representing the relationships among the three clone libraries from each enrichment culture. Grey branches label clone libraries from cultures with lower SMAs while black branches represent libraries from cultures with higher SMAs. The dendrogram also uses 88% sequence similarity to group sequences and so is based on genus relationships not species.

Community analysis was performed on *mcrA* clone libraries from two separate DNA extractions and one RNA extraction. Including all three libraries (2 DNA and 1 cDNA), 245–285 *mcrA* sequences were analysed from each culture (Fig. 4A). The *mcrA* sequences were assigned to methanogen genera using BLAST and the sequence similarity recommended by Steinberg and Regan (2008) (Fig. 4A). A dendrogram was generated showing the relationships among the clone libraries using the Sørenson similarity coefficient (Fig. 4B).

## Discussion

In anaerobic wastewater treatment, methanogens are critically important, serving as both the final step in organic degradation and the source of CH_4_. Therefore, studies of methanogen dynamics can provide valuable information for the development and monitoring of this form of biotechnology.

The enrichment cultures used in these analyses were fed with H_2_/CO_2_ as the primary substrates. Thus, it was expected that hydrogenotrophic methanogens would dominate and analysis of the *mcrA* clone libraries (Fig. 4) generally supported this expectation. The exception was that *mcrA* sequences from *Methanosaeta*, acetoclastic methanogens adapated to low acetate concentrations (Jetten *et al*., 1990), represented about 60% of the clones in R4 in one set of analyses, but these contributed to only ∼ 5% of the *mcrA* transcripts. The low abundance and/or *mcrA* transcript activity of acetoclastic methanogens were probably the major reasons why the SMA analysis with acetate (although limited) was below detection. Using H_2_/CO_2_ also limited the necessity of methanogens obtaining H_2_ from syntrophic acetogens which contributed to the SMA using propionate also being below detection. However, the SMA values measured herein for H_2_/CO_2_ (Fig. 1) were within the previously reported range (30–1500 ml CH_4_ g^−1^ VSS-h^−1^ for pure cultures) for strictly anaerobic cultures (Pavlostathis and Giraldo-Gomez, 1991).

The qPCR results indicated a significant correlation between the abundance of primarily hydrogenotrophic methanogens (*mcrA* copy number) and H_2_/CO_2_ SMA values (Fig. 3). This finding complements previous studies which linked *mcrA* gene copy number to methane flux (Traversi *et al*., 2012). However, the qRT-PCR results did not demonstrate a correlation between *mcrA* transcripts and SMA values. This is in contrast to results found by Munk and colleagues (2012) who found a correlation between methane productivity and the concentration of *mcrA* transcripts. These authors did not use a SMA approach to estimate methane production rates. The results in the present study indicated that the number of methanogens present was more important for the rates of methane production in these H_2_/CO_2_ enrichment cultures. This finding is encouraging in that it indicates that qPCR of *mcrA*, which can be performed within a 24-h time frame, provides information which correlates with SMA, a 2-day to 1-week standard method for determining the activity of anaerobic biomass.

While our values for *mcrA* genes and transcripts were higher than those in peat reported in a study by Freitag and Prosser (2009), this result was expected because of the H_2_/CO_2_ enrichment of the bioreactors for hydrogenotrophic methanogens. However, the previous study did not detect the same strong correlation between *mcrA* gene abundance and measurements of methane flux (Freitag and Prosser, 2009). Instead, transcript to gene copy ratios showed the best correlation with methane production (r^2^ = 0.79, Freitag and Prosser, 2009), but transcript to gene copy ratios for *mcrA* did not correlate with SMA in this study. This difference may be due to several factors including sample type, the diversity of the methanogens in each environment and methods of measurement for methane production.

The variation in the qPCR and qRT-PCR results from each enrichment culture across the three sampling dates (Fig. 2A and B) may have been due to the fact that the biomass samples were not collected at any specific time of day, especially in reference to the daily pulse feeding and biomass wasting. Still, the trend (abundance of *mcrA* genes in R1 and R3 > R2 and R4) across all three dates was clearly the same, and the correlation of the mean values was significant. qPCR and qRT-PCR assays temporally represent a ‘snapshot’ of methanogen abundance, while SMA assays take much longer to complete. Therefore, it made sense to use multiple extractions over time to generate qPCR results for comparison.

Results for qPCR and qRT-PCR were normalized to ng of DNA or RNA respectively. In a similar study with peat, Freitag and Prosser (2009), used both nanogram of nucleic acids and gram of soil. Although it would have been possible to normalize to VSS, a measure of the organic content, or millilitre of culture, the respective nucleic acids were chosen for several reasons. VSS measures all organic content including recalcitrant organic substrates and dead organisms, and thus, the actual active biomass component could have varied widely among the samples. The VSS for each culture ranged from 162 mg VSS l^−1^ in R1 to 515 mg VSS l^−1^ in R2, with VSS values in R3 and R4 falling in between. Therefore, 1 ml of biomass from each reactor could have contained vastly different amounts of organic compounds, and although VSS is not an ideal measure of active biomass, using equal amounts of samples with wide disparity in VSS could represent variation in the abundance of methanogens as well. Using nucleic acids for normalization allowed us to calculate *mcrA* genes or transcripts as a proportion of the DNA or RNA extracted from the sample, making comparison among bioreactors as straightforward as possible.

Other possible explanations for the similar SMA results from cultures R1 and R3 or R2 and R4 were that the methanogens in these cultures were alike or dominated by a particular species, or that the active methanogen populations in cultures with similar SMAs were comparable. We performed community analysis on methanogens in order to rule out these possibilities. After analysing the communities from each culture two ways (Fig. 4A and B), we found no correlation between the community structure of the methanogens in the cultures and SMA values. Therefore, based on our analyses, community structure was not related to methane production rates in these cultures.

Variation among methanogen transcription and translation rates for *mcrA*, as well as the half-life and stability of the mRNA and the resulting protein, may all have affected the outcomes of this study; however, very little of these data is available for methanogen genera. Furthermore, while *mcrA* has been demonstrated to be a valuable gene for use in the investigation of methanogens in the environment, the data obtained from PCR-based methods using primers for *mcrA* are subject to biases inherent in the process (von Wintzingerode *et al*., 1997). However, the primer set designed by Luton and colleagues (2002) has previously been shown to consistently amplify *mcrA* from a wide range of methanogen genera, making the set a sound choice for the examination of methanogens in environmental samples (Luton *et al*., 2002; Banning *et al*., 2005; Juottonen *et al*., 2006). Further physiological information about methanogens and *mcrA* would be useful for interpreting these data as the link between genetic differences in *mcrA* and MCR activity has not been explored.

In summary, the data from this study may be used to better understand methanogenic community structure in anaerobic digesters even though the enrichment process favoured hydrogenotrophic methanogens. Recent papers have indicated the importance of hydrogenotrophic methanogens in anaerobic digesters under some conditions (Kampmann *et al*., 2012; Sundberg *et al*., 2013). Quantification of *mcrA* genes was correlated with SMA values, and therefore, qPCR assays could be a valuable, time saving method for monitoring and assessing anaerobic biomass. Future studies that include lab-scale and industrial-scale digester biomass containing a both hydrogenotrophic and acetoclastic methanogens will be performed to assess this method for wider application. We report a significant correlation between the abundance of *mcrA* gene copies and SMA results. We include analysis of *mcrA* DNA and cDNA clone libraries from each of the bioreactors in order to rule out the influence of similarities among methanogen community structure on these results. These results suggest that SMA assays of biomass activity may be replaced by a faster method, qPCR of *mcrA*.

## Experimental procedures

### Sample sources

Four continuously mixed 2 l bioreactors (R1, R2, R3, R4) were maintained at 35°C in the Civil, Construction and Environmental Engineering Department of Marquette University (see Schauer-Gimenez *et al*., 2010, for additional details). All reactors were sparged with 50:50 H_2_:CO_2_ once a day for approximately 3 min. The cultures were fed H_2_ because its conversion to CH_4_ can be one of many rate-limiting steps in the degradation of complex wastes/substrates. Additionally, R3 and R4 received a measured volume of approximately 80 mg O_2_ (6% of the hydrogen COD) once a day because increased SMA with light aeration has been previously observed and reported (e.g. Zitomer and Shrout, 1998). R2 and R4 also received 84 mg of glucose daily in addition to H_2_/CO_2_, and therefore, fermentation products, including acetate, were ostensibly present.

### SMA assays

Acetate and propionate SMA assays were performed once and H_2_/CO_2_ (4:1 v/v H_2_:CO_2_) assays were performed 13 times over the course of a year and a half. They were conducted in triplicate at 35°C under anaerobic conditions in 160 ml serum bottles with 25 ml of culture. The VSS concentration was determined at the beginning and end of activity tests, and the average of the two values was employed for calculations [American Public Health Association (APHA) *et al*., 1998 ]. The serum bottles were sparged with gas, sealed, and then 100 ml of the H_2_:CO_2_ gas blend was injected. Bottle headspace volume was measured at ambient pressure for 1–5 days by inserting the needle of a glass syringe. Syringe content was re-injected into the serum bottle after volume measurement. The maximum methane production rate (ml CH_4_ g^−1^ VSS-h^−1^) was determined as described by Coates and colleagues (1996).

### Nucleic acid (DNA and RNA) extraction

Nucleic acids were extracted from biomass samples immediately after their collection. RNA was extracted using the RNA Powersoil RNA Total RNA Isolation kit (MOBIO, Carlsbad, CA, USA) according to the manufacturer's standard protocol. DNA was extracted by using one of two kits: DNA Powersoil® DNA Isolation kit using alternative lysis protocol (DNAI clone libraries only) or the DNA Elution Accessory kit (MOBIO). DNA samples were purified using the Powerclean® DNA Cleanup Kit (MOBIO). RNA samples were treated with Rnase-free Dnase (Rnase-free Dnase Set, Qiagen, Valencia, CA, USA) and purified using the Rneasy® Mini Kit (Qiagen). After purification, samples were checked for integrity on agarose gels (1.5% w/v) and then quantified using a spectrophotometer (Nanodrop ND-1000, ThermoScientific, Waltham, MA, USA).

### RT-PCR

RT-PCR was performed on 645–1900 ng of RNA from each digester using the iScript Select cDNA Synthesis Kit (Biorad, Hercules, CA, USA) using 500 nM of the *mcrA* reverse primer Luton and colleagues (2002). A no-template control was included in each run and no-RT controls were included for each sample. The RT reaction conditions were as follows: 42°C for 1 h 30 min and then 85°C for 5 min.

### PCR

The primer pair designed by Luton and colleagues (2002) (mcrF 5′-GGTGGTGTMGGATTCACACARTAYGCWACAGC-3′; mcrR 5′-TTCATTGCRTAGTTWGGRTAGTT-3′) was used for PCR resulting in a ∼ 460 bp product of *mcrA*, the gene encoding the α subunit of MCR. The final component concentrations per 50 μl PCR reaction were as follows: 100 nM each primer, 0.2 mM dNTPs, 1X Colorless GoTaq Reaction Buffer which contained 1.5 mM MgCl_2_ (Promega, Madison, WI, USA) and 1.25U GoTaq polymerase (Promega). Template concentrations were approximately 100 ng per reaction tube. The PCR conditions were as follows: 95°C (5 min), 35 cycles of 95°C (1 min), 49°C (1 min) and 72°C (3 min), and a final extension 72°C (10 min). The programme included a slow ramp in temperature (0.1°C s^−1^) between the annealing and extension steps of the first five cycles of the protocol to assist in the initial formation of product because of the degenerate nature of the primers (Luton *et al*., 2002).

### Cloning

Clone libraries were constructed by ligating the *mcrA* PCR products into the pCR 2.1-TOPO® vector and then transformation into One Shot TOP10 chemically competent *Escherichia coli* using the TOPO TA® cloning kit according to the manufacturer's instructions (Invitrogen, Carlsbad, CA, USA) and identified by blue-white screening of the transformants (Sambrook and Russell, 2001). Randomly selected white colonies were used for direct PCR with the vector-specific primers PUCF (5'-GTAAAACGACGGCCAG-3') and PUCR (5'-CAGGAAACAGCTATGAC-3') (Invitrogen). The 50 μl final volume PCR reaction component concentrations were as described above and conditions for the PUC primers were: denaturing at 94°C (1 min), annealing at 55°C (1 min) and extension at 72°C (1 min), and a final extension at 72°C (10 min).

### Community analysis

Clones from the first DNA extraction (DNA I library) were subjected to restriction fragment length polymorphism (RFLP) analysis with *MspI*, *RsaI and Taq^α^I* (New England Biolabs, Ipswich, MA, USA). Clones with unique RFLP patterns and all clones from DNA II and the cDNA library were purified using Qiaquick PCR Purification Kit (Qiagen), normalized to a concentration of 50 ng μl^−1^ and sequenced at the University of Chicago Cancer Research Center DNA Sequencing Facility. The forward and reverse sequences were assembled into consensus sequences using the ContigExpress tool in VectorNTI. Residual vector sequence was removed from the consensus sequences using a software programme that utilized VecScreen (National Center for Biotechnology Information). BLAST (blastn) searches were conducted with the *mcrA* sequences to determine their relationship to reference *mcrA* sequences in GenBank®.

The sequences were aligned using CLUSTALW (Thompson *et al*., 2013) and then analysed in mothur (sequence similarity cut-off = 0.12) (Schloss *et al*., 2009). A dendrogram representing the relationship among the sequence libraries was generated in mothur using a distance matrix based on the Sørenson index, which describes the similarity of communities based on the presence and absence of particular members and the observed richness.

### qPCR

qPCR was performed in triplicate according to the recommendations by Smith and colleagues (2009) and Smith and Osborn (2009) except for the standard curve (see below), and the Minimum Information for Publication of Quantitative Real-Time PCR Experiments guidelines which were applicable to environmental samples (Bustin *et al*., 2009) using the same primers designed by Luton and colleagues (2002) (Vianna *et al*., 2006; Goffredi *et al*., 2008; Freitag and Prosser, 2009; Freitag *et al*., 2010). The final qPCR mix per 25 μl reaction was as follows: 1X iQ SYBR® Green Supermix reaction buffer containing dNTPS, iTaq DNA polymerase and 3 mM MgCl_2_ (Biorad); 750 nM mcrF and mcrR; and template DNA (0.3–1 ng) or cDNA (1 μl of RT-PCR reaction or more, when needed, to bring up to the final 25 μl). Each qPCR run included a no-template control and the no-RT controls from the RT reactions. The qPCR reactions were performed with the Biorad MyIQ Single-Color Real-Time PCR Detection System using the following programme: initial denaturation at 95°C (10 min), 45 cycles of 95°C (30 s) and 58.5°C (1 min), and a final extension of 7 min at 72°C, followed by a disassociation curve programme to check for product specificity. Products from initial runs were also examined for specificity using 1.5% agarose gels. Starting quantity amounts and threshold cycle values were calculated using the MyiQ optical system software version 1.0. Gene copy numbers and transcripts were calculated from starting quantities provided by the MyiQ software based on the molecular weights of the ∼ 460 bp of *mcrA* DNA or RNA. Transcript to gene copy ratios were normalized using total nucleic acid concentrations (DNA and RNA combined) from each extraction.

### Standards

qPCR standards used in all runs were created using pooled *mcrA* DNA clones (50 ng each) representing a broad spectrum of *mcrA* sequences representative of methanogen genera commonly seen in anaerobic digesters: *Methanospirillum*, *Methanobacterium*, *Methanosaeta*, *Methanoculleus*, *Methanobrevibacter* (Steinberg and Regan, 2008). Their nucleotide sequences can be found in GenBank® under accession numbers HM800527-528, HM800531, HM800534-536, HM800542, HM800547, HM800549, HM800560, HM80072, HM800574, HM800581 and HM800611.

### Statistical analysis of qPCR results

Linear regression with the qPCR or qRT-PCR results or transcript to gene copy ratios and SMA values was performed using R to calculate r^2^ and *P*-values (R Core Team, 2012). Values were plotted with a trend line for visual analysis.

### Nucleotide sequence accession numbers

All nucleotide sequences can be found in the GenBank® database under accession numbers HM800526 through HM800637, HM80666 through HM80695 and JF460039 through JF460714.
